# Isolation, purification, structural analysis and coagulatory activity of water-soluble polysaccharides from *Ligustrum lucidum* Ait flowers

**DOI:** 10.1186/s13065-017-0332-y

**Published:** 2017-10-04

**Authors:** Zhenhua Yin, Wei Zhang, Juanjuan Zhang, Wenyi Kang

**Affiliations:** 1 Huanghe Science and Technology College, Zhengzhou, 450063 China; 2 Zhengzhou City Key Laboratory of Medicinal Resources Research, Zhengzhou, 450063 China

**Keywords:** *Ligustrum lucidum* Ait flowers, Polysaccharides, Coagulatory activity

## Abstract

In this study, *Ligustrum lucidum* flowers as raw material, the extraction, isolation and coagulatory activity of polysaccharides were carried out for the first time. The crude polysaccharide was obtained by hot water extraction and ethanol precipitation, and preliminarily purified by Sevage method and D101 macroporous resin. Then the polysaccharide was further purified by DEAE-52 cellulose and Sephadex G-100 column chromatography, respectively. The structural characteristics were detected by LC, GC, FT-IR and NMR. Furthermore, the coagulatory activity of the polysaccharides were investigated by APTT, TT, PT and FIB assays in vitro. The results demonstrated that four polysaccharides were isolated from flowers of *L. lucidum*, named as LLP-1a, LLP-1b, LLP-2 and LLP-3, and the yields were 0.039, 0.0054, 0.0055 and 0.017%, respectively based on the weight of the dried flowers. The four polysaccharides components were free of nucleic acids and proteins, and their average molecular weights were 25,912, 64,919, 3,940,246 and 2,975,091 g/mol, respectively. The monosaccharide compositions of LLp-1a were l-rhamnose, l-arabinose, d-xylose, d-glucose and d-galactose (molar ratio of 3.16: 2.46: 1.00: 7.27: 4.22). Only d-galactose was detected from LLp-1b. LLp-2 was composed of l-arabinose, d-glucose and d-galactose (molar ratio of 1.28:1.32:1.00). LLp-3 was composed of l-rhamnose, l-arabinose, d-xylose, d-glucose and d-galactose (molar ratio of 5.85: 2.21: 2.23: 1.00: 2.25). Coagulation assays indicated that LLp-1a and LLp-3 had good anticoagulant effect in vitro, while LLp-1b showed procoagulant activity.

## Background

*Ligustrum lucidum*, belonging to *Ligustrum* genus, a flowering plant in the Oleaceae family, is native to the south of the Yangtze River to South China, southwest provinces and autonomous regions, Northwest distribution to Shanxi, Gansu, and naturalized in several other countries including India, Nepal and Korea [1]. At present, “Chinese Materia Medica” records the fruits, leaves, barks and roots of *L. lucidum*. Its fruit is often called “Nüzhenzi”, as a traditional Chinese medicine. There are more studies on its chemical constituents and pharmacological effects [2–6], but the research on flowers is relatively few, only some reports have studied the chemical composition and pharmacological activity, for example, Yang et al. [7] characterized the chemical composition of essential oil from the its flowers. Long et al. [8], Wang and Hou [9] studied the chemical constituents in flowers, sterols, flavonoids and alcohols were isolated from flowers. Zhang [10] found the anthocyanins in flowers had strong antioxidant activity in vitro. Yao et al. found the total flavonoids in flowers had the activities on scavenging DPPH free radicals and nitrite [11, 12]. About polysaccharides of *L. lucidum*, only Shi et al., have studied the polysaccharides from its fruit, found the polysaccharide could markedly improve the immune functions of hydrocortisone-induced immunosuppressed model mouse [13].

However, the polysaccharides in flowers are still uncertain without a clear theoretical evidence. Hence, the preliminary identification of the compositions of flowers polysaccharides would be significant and advantageous to be studied for further illustration of their potential bioactivities.

Thrombosis involves local blood clotting of the vascular system that often leads to serious health-related diseases such as heart attacks and strokes. The risk factors for thrombosis are abnormal hyperlipid, hyperglycemia, elevated plasma fibrinogen, high blood pressure and cancer, these thrombotic diseases, have become the primary causes of death and their incidence has been increasing annually [14, 15]. Therefore, effective antithrombotic drugs are urgently needed.

It is well known that polysaccharides have many bio-activities, such as antioxidant [16], laxative [17], hypoglycemic [18], immunomodulating activity [19]. In recent years, the research on the coagulation activity of polysaccharides has also been welcomed by many scholars [20, 21]. Up to now, there is no investigation report on the coagulation active ingredient of *L. lucidum* flowers.

Based on the above analysis, the objective of this research was to extract and purify the bioactive polysaccharides in flowers of *L. lucidum* with coagulation activity (Due to the large molecular weight, poor solubility limited sample size of polysaccharides, we only carried out coagulation activity in vitro), which could provide theoretical basis for its further application, and might expand the possibility to find better coagulation drug.

## Methods

### Plant material

The flowers of *L. lucidum* were collected in April 2015 from Guiyang City, Guizhou Province, and were identfied by Prof. Qian-jun Zhang. The voucher specimens were deposited in the herbarium of Huanghe Science and Technology College.

### Animals

Male rabbit (2.0–2.5 kg), was purchased from the Experimental Animal Center of Henan Province (Zhengzhou, Henan, China, No: 14-3-7).

### Reagents

Dextrans with different Mw (T-40, T-64, T-150, T-250 and T-500) were purchased from Sigma-aldrich. Monosaccharide standards including L-rhamnose (Rha), l-arabinose (Ara), d-xylose (Xyl), d-mannose (Man),d-glucose (Glc), d-galactose (Gal) were obtained from Dr. Ehrenstorfer GmbH Co. (Germany). Sephadex G-100 and DEAE-52 cellulose gel were purchased from GE Healthcare Bio-Scinence (Germany). Trifluoroacetic acid (TFA, standard for GC, > 99.8%) was purchased from Aladdin (Shanghai, China). Hydroxylammonium chloride (guarantee reagent) and pyridine were purchased from Tianjin Kemiou chemical reagent co., LTD. Injection breviscapine (Lot: 15141005) was obtained from Hang Sheng Pharmaceutical Co., Ltd. (Hunan, China). Yunanbaioyao (Lot: ZGA1604) was obtained from Yunnan Baiyao Group Co., Ltd. (Yunan, China). APTT (Lot: 1121911), TT (Lot: 121168), PT (Lot: 105295) and FIB (Lot: 132107) assay kits were purchased from Shanghai Sun Biotech Co., Ltd (Shanghai, China).

### Extraction, purification of the crude polysaccharides

The dried flowers of *L. lucidum* (475 g) were crushed and refluxed with petroleum ether twice for 2 h to remove liposoluble constituents, and the polar constituents were removed by the soaking of 70% ethanol for 3 days. The degreased flowers were extracted twice by ultrapure water (W/V 1:12) that prepared with a Mill-Q water purification system (Merck Millipore Germany) at 85 ± 0.5 °C for 5 and 4 h. The extracting solution were merged, filtered and concentrated with rotatory evaporation till a quarter of the total volume. The concentrated solution was mixed with alcohol (2.8 vol) to obtain the crude polysaccharide.

The protein present was removed by Sevage method [22], and due to the dark color, D101 macroporous resin was applied to decolorize crude polysaccharide, followed by centrifugation (6000 rpm for 15 min at 4 °C) and alcohol precipitation (2.8 vol). Then the refined polysaccharide was redissolved in water and dialyzed with dialysis bag (Molecular weight cut-off 8000–14,000 Da) for 24 h in distilled water and another 12 h in ultra-pure water. Finally, the dialyzed polysaccharide solution was dehydrated by freeze-drying using LL-1500 Freeze Dryer (Thermo) to obtain refined polysaccharide.

The refined polysaccharide was further purified by DEAE-52 cellulose gel (2.5 × 60 cm) and was eluted sequentially with 0.0, 0.1, 0.2 and 0.3 mol/L NaCl. The purified fraction showed three main peaks (LL-1, LL-2 and LL-3), after that the Sephadex G-100 column (1.5 × 100 cm) was used to fractionate the three fractions. LL-1 fractionated into two polysaccharides, named as LLp-1a, and LLp-1b, respectively. LL-2 fractionated one polysaccharide, named as LLp-2, and LL-3 fractionated into one polysaccharide, named as LLp-3.

### UV–Vis spectrophotometer analysis

The freeze-dried four polysaccharides were mixed with ultrapure water to make concentration of 0.1 mg/mL solution for the analysis. The spectrum was scanned from 200 to 760 nm by Hitachi U-4100 UV–Vis spectrophotometer.

### Determination of the average molecular weight and monosaccharide composition

The average molecular weights of four polysaccharides (LLp-1a, LLp-1b, LLp-2 and LLp-3) were determined by liquid chromatograph (Waters) equipped with an differential refraction detector and TSK G4000P W_XL_ chromatographic column (7.8 mm × 300 mm × 17 μm, Japanese east cao co., LTD), and the polysaccharide solutions 10 μL, previously filtered through a membrane (0.22 μm, Millipore), was injected at a concentration of 1 mg/mL, and run with Watsons purified water at 1.0 mL/min as mobile phase. The standard curve was established using using T-40, T-64, T-150, T-250 and T-500 as standard dextrans.

Freeze-dried four polysaccharides (10 mg) were hydrolyzed with 2 mL 2 mol/L of trifluoroacetic acid (TFA) in oven for 3 h at 110 °C in nitrogen sealed ampoule bottles. The soluble fraction was evaporated to dryness under stream of nitrogen to get hydrolysates. The hydrolysates were incubated with 10 mg hydroxylamine hydrochloride and 0.5 mL pyridine in water bath for 30 min at 90 °C, and then were acetylated with 0.5 mL Ac_2_O at 90 °C for 30 min. The acetylates were filtered through a membrane and readied for GC analysis. GC was used to determine the monosaccharide peak area. GC analysis was equipped with a HP capillary column (30 m × 0.35 mm, 0.25 μm) and a FID detector, and nitrogen was used as carriergas (2 mL/min). The program was isothermal at 100 °C, hold for 1 min, with a temperature gradient of 4 °C/min up to a final temperature of 240 °C, hold for 10 min. The injector temperature was 250 °C, and detector temperature 280 °C. l-rhamnose, l-arabinose, d-xylose, d-mannose, d-glucose, d-galactose were also derivatized as standard.

### FT-IR analysis

1 mg of freeze-dried four polysaccharides were mixed with 150 mg of dried potassium bromide (KBr), and pressed into disk for the analysis. The IR spectrum was recorded in the range of 400–4000/cm on a Thermo Scientific Nicolet iS5 Fourier transform infrared spectroscopy (Thermo Electron, USA).

### NMR spectral analysis

The samples (20 mg) were freeze-dried with 500 μL D_2_O (99.9%) three times before dissolution in 500 μL D_2_O (99.9%), finally transferred into 5-mm NMR tube. The one-dimensional NMR spectra (^1^H-NMR and ^13^C-NMR) were conducted on Bruker Avanced III 400 MHz equipment (Billerica, MA, USA). The chemical shifts of ^1^H-NMR spectra were calibrated with reference to D_2_O, used as an internal standard at 4.70 ppm.

### Coagulation activity test

The coagulation activity of four polysaccharides was evaluated by activated APTT, TT, PT and FIB assays in vitro.

#### Preparation of sample and positive control

Weigh a certain amount of polysaccharide dissolved in a certain volume solvent (anhydrous ethanol: 1,2-propylene glycol: physiological saline = 1:1:3, volume ratio), and configured to a concentration of 5 mg/mL solution. Breviscapine was configured to a concentration of 13.33 mg/mL, and the concentration of Yunnanbaiyao was 40 mg/mL.

#### Preparation of plasma

Blood samples were taken at the ear vein of rabbits, and added to centrifuge tubes containing 0.4 mL, 0.109 mol/L of sodium citrate, the mixture was centrifuged to separate the supernatant at 3000 rpm for 15 min.

#### APTT assay

25 μL polysaccharide solution was added to the test cup, and then add 100 μL of plasma and 100 μL of APTT reagent pre-warmed at 37 °C in the test cup. The above reaction solution was incubated at 37 °C for 5 min, and then 100 μL of 0.025 mol/L CaCl_2_ solution at 37 °C pre-temperature was added to record the coagulation time by HF6000-4 semi-automatic coagulation analyzer, the time was the APTT value.

#### TT assay

50 μL of polysaccharide solutions was added to the test cup, and then 200 μL of plasma was added to the test cup. After incubation at 37 °C for 3 min, 200 μL PT reagent was added to record the coagulation time by HF6000-4 semi-automatic coagulation analyzer, the time was the TT value.

#### PT assay

25 μL of polysaccharide solutions was added to the test cup, and then 100 μL of plasma was added to the test cup. After incubation at 37 °C for 3 min, 200 μL 37 °C pre-warmed PT reagent was added to record the coagulation time by HF6000-4 semi-automatic coagulation analyzer, the time was the PT value.

#### FIB assay

First of all, according to the requirements of specification to draw the standard curve, and then sample determination. Take 200 μL of plasma and 100 μL of polysaccharide solutions, then add 700 μL of buffer, 200 μL of the above mixture was taken and incubated at 37 °C for 3 min. Finally, 100 μL thrombin solution was added to the above mixture to record the content of fibrinogen, the content was FIB value.

For the four methods, solvent was used as blank control, breviscapine and Yunnanbaiyao were used as positive control.

## Results and discussion

### Polysaccharide isolation and purification

**Fig. 1:**
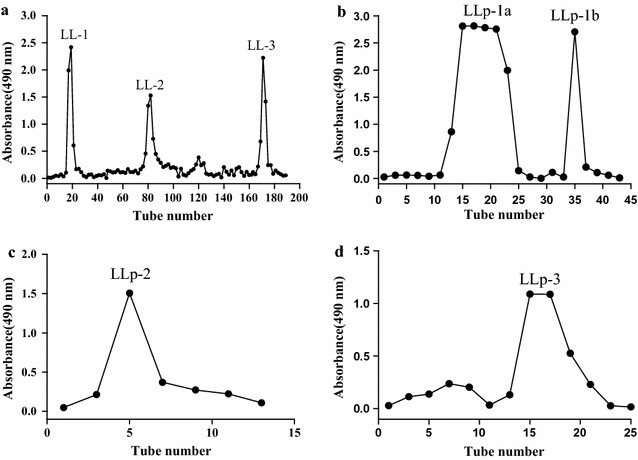
Elution curve of crude polysaccharide by DEAE-52 cellulose column chromatography (**a**), elution curve of LL-1 on Sephadex G-100 column (**b**), elution curve of LL-2 on Sephadex G-100 column (**c**), elution curve of LL-3 on Sephadex G-100 column (**d**)

After removing the protein and pigment, the refined polysaccharides were preliminary purified by DEAE-52 cellulose column chromatography, three main polysaccharide fractions were obtained, named LL-1 eluted with 0.1 mol/L NaCl, LL-2 eluted with 0.2 mol/L NaCl and LL-3 eluted with 0.3 mol/L NaCl, respectively (Fig. 1a). The three polysaccharide fractions isolated by DEAE-52 were further isolated and purified by Sephadex G-100 column chromatography. Finally, two polysaccharides were isolated from LL-1, named as LLp-1a (183.7 mg) and LLp-1b (26 mg) (Fig. 1b), LL-2 and LL-3 eluted two polysaccharides, respectively, named as LLp-2 (25.5 mg) (Fig. 1c) and LLp-3 (83 mg) (Fig. 1d).

### UV–Vis spectroscopy analysis

**Fig. 2:**
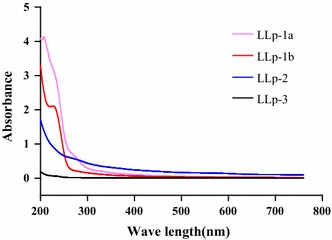
UV-Vis spectra full-wavelength scanning curves of LLp-1a, LLp-1b, LLp-2 and LLp-3

Nucleic acids and proteins have UV absorption at 260 and 280 nm wavelengths, so, UV–visible full-wavelength scanning was used to determine whether polysaccharide solution contained protein and nucleic acid. The scanning result of the four polysaccharides was shown in Fig. 2. The four polysaccharides had no significant absorption peak at 260 and 280 nm, which indicated that the four polysaccharides were free of nucleic acid and protein.

### Molecular weight analysis

Most of the polysaccharides were obtained with water extract alcohol precipitation, and the extracted polysaccharides were mostly viscous and unstable colloidal solution. The relative molecular mass of the components contained in the colloidal solution was different, and the pharmacological activity of polysaccharides with different relative molecular weights was quite different, which brought great difficulties for the quality control and further development and utilization of polysaccharide. Therefore, it was necessary to screen the polysaccharides of different molecular segments and determine their molecular weight [23]. At present, the molecular weight of polysaccharides could be measured by several techniques, such as vapor pressure method, end-based analysis, osmotic pressure, viscosity method, high performance liquid chromatography, high performance size-exclusion chromatography (HPSEC) [24], multiple-angle laser light scattering (MALLS) [25], and high-performance gel permeation chromatography (HPGPC) [26, 27]. In our study, the molecular weights were measured by LC equipped with a refractive index detector, with the dextran standards (T-40, T-64, T-150, T-250, and T-500) used for the calibration curve. The equation of the standard curve was: Log^*Mw*^ = − 0.539*t* + 9.700 (Note: *Mw* represents molecular weight, while *t* represents retention time) with a correlation coefficient of 0.988. As it is shown in Table 1, the average molecular weight of LLp-1a, LLp-1b, LLp-2, LLp-3 were estimated to be 25,912, 64,919, 3,940,246 and 2,975,091 g/mol, respectively.

**Table 1:** Molecular weight of polysaccharides form *Ligustrum lucidum* Ait flowers

Polysaccharide	T (min)	LgMw	Mw	Average Mw (g/mol)
LLp-1a	9.796	4.413	25,882	25,912
	9.794	4.414	25,941	
LLp-1b	9.091	4.794	62,230	64,919
	9.023	4.83	67,608	
LLp-2	5.762	6.591	3,899,420	3,940,246
	5.745	6.6	3,981,071	
LLp-3	5.978	6.474	2,978,516	2,975,091
	5.979	6.473	2,971,666	

### Analysis of monosaccharide composition

**Fig. 3:**
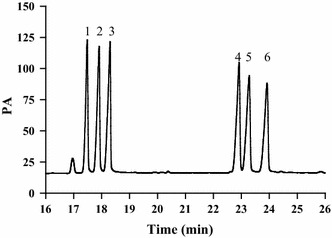
Gas chromatograms of standard monosaccharide mixture solution (1) l-rhamnose (Rha) (2) l-arabinose (Ara) (3) d-xylose (Xyl) (4) d-mannose (Man) (5) d-glucose (Glu) (6) d-galactose (Gal)

**Fig. 4:**
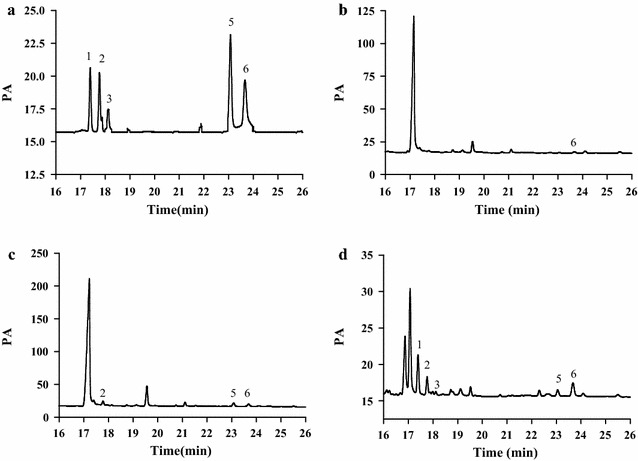
Gas chromatograms of the monosaccharide compositions of polysaccharides LLp-1a (**a**), LLp-1b (**b**), LLp-2 (**c**) and LLp-3 (**d**) from *L. lucidum* flowers

Previous studies have shown that the strong biological activity of polysaccharides was strongly related to monosaccharide compositions [28], and the monosaccharide composition of polysaccharides played an important role in further analyzing its physicochemical properties, structure and structure-biological activity. At present, there were many ways to determine the monosaccharide composition, including high performance liquid chromatography [29], reversed-phase high performance liquid chromatography (HPLC) after pre-column derivatization [30], high-performance thin-layer chromatography [31], gas chromatography (GC) [32], high-performance anion-exchange chromatography [33], high performance capillary electrophoresis [34]. In our study, the monosaccharide compositions were measured by GC with good sensitivity, and monosaccharide composition was estimated by comparing retention time (RT). The results were shown Figs. 3, 4. As could be seen from the figures, the peaks of all monosaccharides were sharp and symmetrical. Compared with the standard monosaccharides (Fig. 3), the peaks of the LLp-1a derivatives were identified as l-rhamnose, l-arabinose, d-xylose, d-glucose, d-galactose, LLp-1a was a heteropolysaccharide and in a molar ratio of 3.16: 2.46:1.00: 7.27: 4.22. Only d-galactose was detected from LLp-1b. The monosaccharide compositions of LLp-2 were l-arabinose, d-glucose and d-galactose, and in a molar ratio of 1.28:1.32:1.00. The monosaccharide compositions of LLp-3 were l-rhamnose, l-arabinose, d-xylose, d-glucose and d-galactose, and in a molar ratio of 5.85: 2.21: 2.23:1.00:2.25.

### FT-IR spectroscopy analysis

**Fig. 5:**
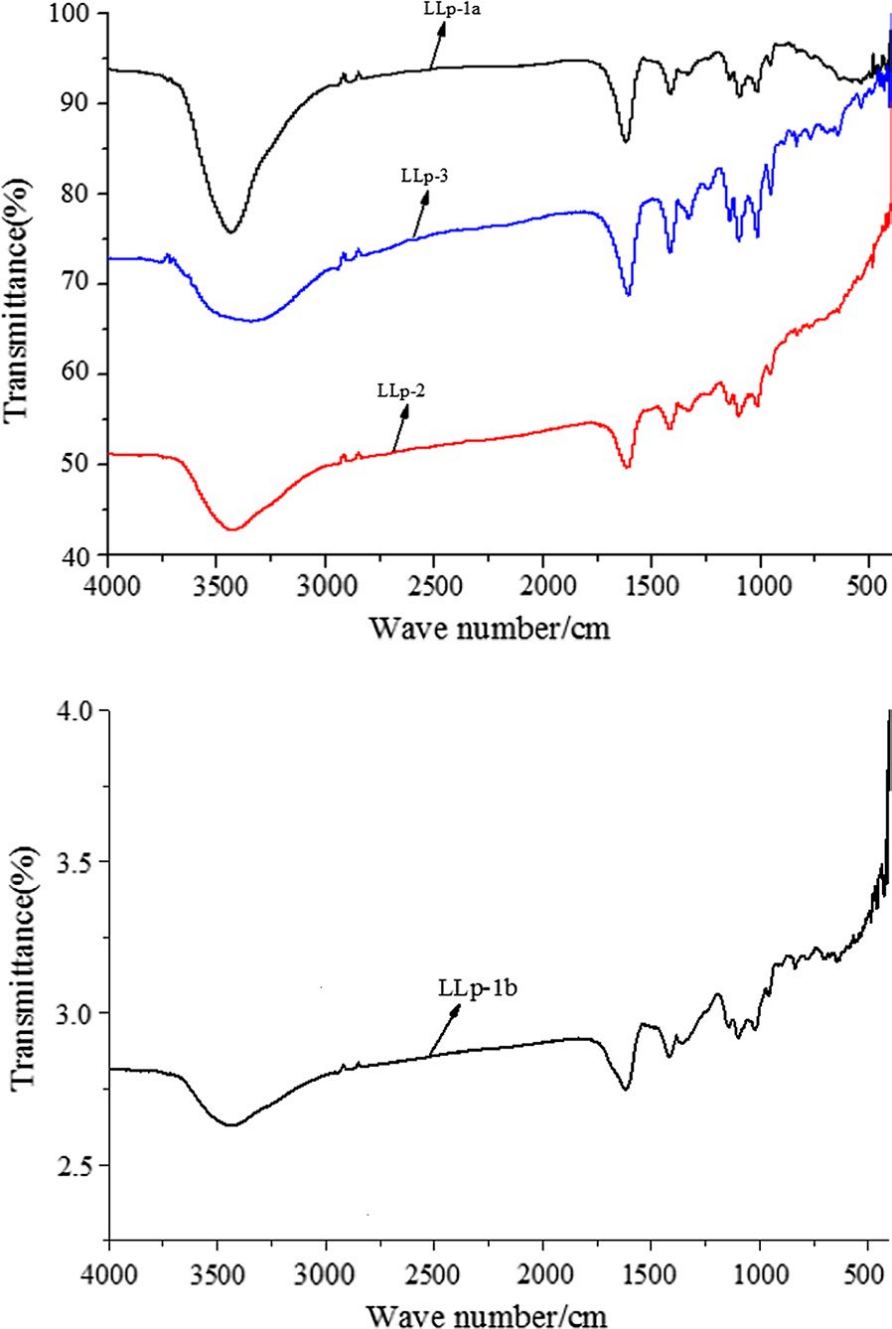
FT-IR spectra of LLp-1a, LLp-1b, LLp-2 and LLp-3

The FT-IR spectroscopys of LLp-1a, LLp-1b, LLp-2 and LLp-3 were recorded at the range of 4000–400/cm (Fig. 5). Obviously, it was showed that the IR spectra of four polysaccharides had a strong characteristic absorption band at 3436, 3425, 3436 and 3346 cm^−1^ for the stretching of hydroxyl, which was common to polysaccharides, then a very weak characteristic absorption appearing at 2947, 2946, 2947 and 1948/cm, respectively, were the absorption peaks of C–H stretching vibration [35]. The strong asymmetrical absorption peak at 1618, 1617, 1617 and 1608/cm, respectively, and weak symmetrical peaks at around 1332–1420/cm were indicative the carboxyl groups and carbonyl groups, which indicated the characteristic IR absorption of uronic acid [36]. According to the study, furanose had two absorption peaks at the range of 1100–1010/cm, and pyranose had three absorption peaks at the range of 1100–1010/cm. Four polysaccharides showed two absorption peaks at 1100–1010/cm, indicating that the four polysaccharides contained furanose rings [37]. Two conformers of carbohydrates, *α*-and *β*-conformers, which depended on the types of end carbon-glucoside bonds, could be distinguished based on the anomeric region-vibrational bands from 950 to 750/cm [38], where around 840/cm corresponds to *α*-conformers, while the *β*-conformers lie around 890/cm [39].

### NMR spectral analysis

**Fig. 6:**
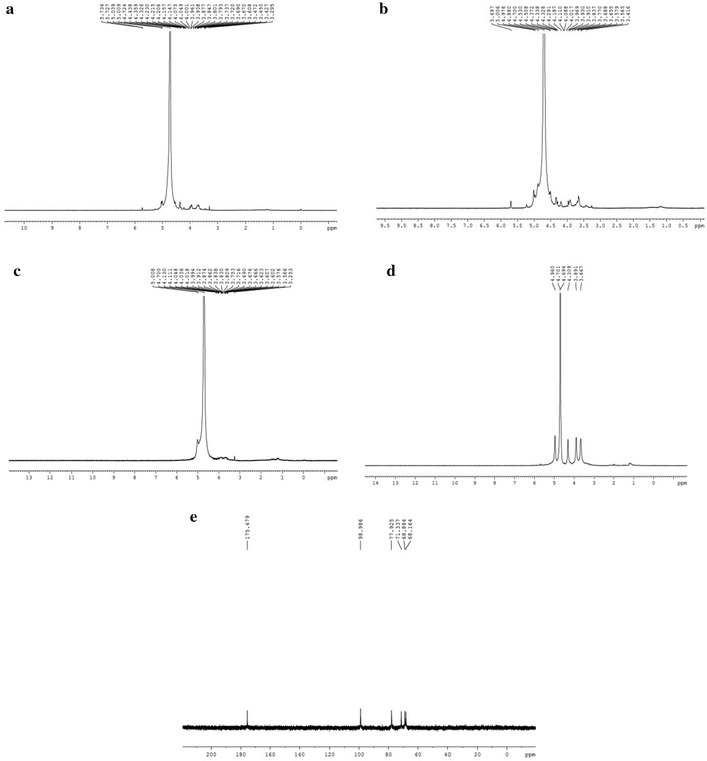
^1^H NMR spectrum of LLp-1a (**a**), LLp-1b (**b**), LLp-2 (**c**) and LLp-3 (**d**), ^13^C NMR spectrum of LLp-3 (**e**)

The ^1^H-NMR spectra of LLp-1a, LLp-1b, LLp-2, LLp-3 and ^13^C-NMR spectra of LLp-3 were shown in Fig. 6, respectively. The ^1^H signal at 4.70 ppm was caused by D_2_H. General speaking, the signals in the region of 5.60–4.90 ppm was assigned to anomeric protons of *α*-anomers, and 4.90–4.30 ppm was assigned to anomeric protons of *β*-anomers, while the region of 4.50–3.00 ppm was contributed to the ring proton region [40]. These data confirmed the backbone had *α*-glycosidic and *β*-glycosidic linkages, which were consistent with the results obtained by FT-IR analysis. The region of 4.50–3.00 ppm were assigned to the H-2 to H-6 protons.

The ^13^C-NMR spectrum of LLp-3 had carboxy carbon signal from 170 to 176 ppm, which illustrated LLp-3 contained uronic acid. Polysaccharide signals generally appeared in the range of 60–110 ppm. Among them, 90–110 ppm for end-based carbon signal, 60–90 ppm for the non-terminal carbon signal. Due to the poor solubility of LLp-1a, LLp-1b and LLp-2, their carbon spectrum signals was not good, but FT-IR spectroscopy analysis indicated that the characteristic IR absorption of uronic acid was existed, which also induced carboxy carbon signal in carbon spectrum, showed the existence of a carboxylic group.

### Coagulation activity in vitro

The effects of polysaccharides on plasma coagulation parameters in vitro including APTT, PT, TT and FIB were assayed and the results were described as follows.

**Fig. 7:**
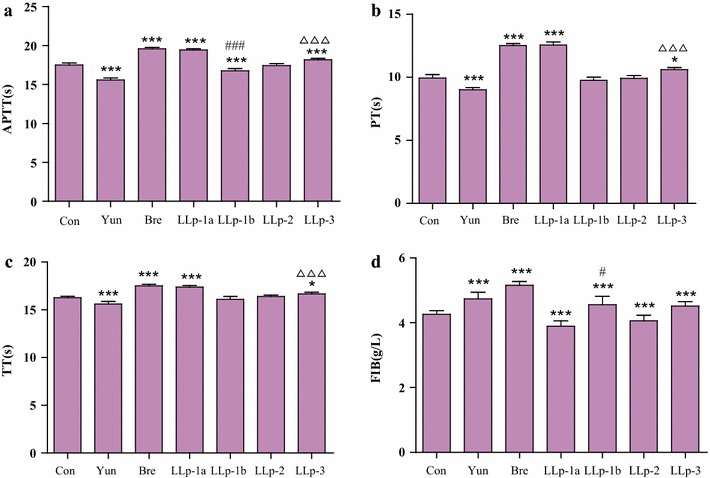
Effects of polysaccharides on plasma coagulation parameters in vitro (**a** APPT; **b** PT; **c** TT; **d** FIB. n = 6). Compared with control group, *** *p* < 0.001 < ** *p* < 0.01 < * *p* < 0.05; Compared with Yunnan Baiyao, ^#*##*^ *p* < 0.001 < ^*##*^ *p* < *0.01* < ^*#*^ *p* < 0.05; Compared with breviscapine, ^△△△^ *p* < 0.001 < ^△△^ *p* < *0.01* < ^△^ *p* < *0.05*

As could be seen in the Fig. 7, compared with the control group, LLp-1a and LLp-3 significantly prolonged APTT, PT and TT (*p* < 0.001 or *p* < 0.05), and the effects of LLp-1a on prolonging APTT, PT and TT were similar to breviscapine as positive control (*p* > 0.05), the effects of LLp-3 were significantly weaker than that of breviscapine (*p* < 0.001). In contrast, compared with the control group, LLp-1b could significantly shorten APTT (*p* < 0.001), the times of LLp-1b on prolonging PT and TT were shorter than that of control group, but longer than that of Yunnanbaiyao as positive control, the effect of LLp-1b was significantly weaker than that of Yunnan Baiyao (*p* < 0.001). For FIB, compared with the control group, LLp-1a significantly reduced FIB content (*p* < 0.001), and LLp-1b and LLp-3 significantly increased FIB content (*p* < 0.001). From the above data comprehensive analysis, we demonstrated that LLp-1a and LLp-3 had good anticoagulant effect, while LLp-1b had procoagulant activity in vitro.

In clinical tests of blood coagulation, several well-established analyses are used to indicate coagulation activity including APTT, PT, TT and FIB. These assays indicate anti-coagulant activity with respect to the intrinsic and extrinsic pathways in the blood coagulation cascade. PT reflects the extrinsic pathway of the coagulation cascade, whilst APTT reflects changes in the intrinsic pathway of the blood, TT is mainly a reflection of the degree of the conversion of fibrinogen into fibrin and is an important index. FIB mainly reflects the content of fibrinogen [41, 42]. In this study, LLp-1a and LLp-3 could prolong APTT and PT, which suggested that the anticoagulant effect of LLp-1a and LLp-3 might be partially due to altered activity of coagulation factors in both extrinsic and intrinsic clotting pathways [42]. LLp-1a and LLp-3 could prolong TT, but LLp-1a significantly reduced FIB content, LLp-3 significantly increased FIB content. These results showed that LLp-1a could benefit hindering fibrin formation. LLp-1b could significantly shorten APTT and increased FIB content, which indicated that its effects were mediated mainly through the intrinsic coagulation pathway and increasing the content of FIB [15].

## Conclusions

In the paper, four polysaccharides were purified from *L. lucidum* flowers by DEAE-52 cellulose and Sephadex G-100 column chromatography, they were free of nucleic acid and protein. The average molecular weights of LLp-1a, LLp-1b, LLp-2 and LLP-3 were 25,912, 64,919, 3,940,246 and 2,975,091 g/mol, respectively, and their monosaccharide compositions were different, which might affect their activities, LLp-1a and LLp-3 had good anticoagulant effect in vitro, while LLp-1b had procoagulant activity in vitro. The further structural analysis were detected by Fourier transform infrared (FT‑IR) spectrometer and nuclear magnetic resonance spectra (NMR). These results implied these polysaccharides had the potential to be developed as natural medicines or health foods with coagulation activity. However, the structure and mechanism of the biological activity of these polysaccharides still need further study.

## Abbreviations

LC: liquid chromatograph
GC: gas chromatography
FT-IR: fourier transform infrared
NMR: nuclear magnetic resonance
ATPP: activated partial thromboplastin time
TT: thrombin time
PT: prothrombin time
FIB: fibrinogen
TFA: trifluoroacetic acid
Rha: l-rhamnose
Ara: l-arabinose
Xyl: d-xylose
Man: d-mannose
Glc: d-glucose
Gal: d-galactose
